# Diagnostic and Management Considerations in a Patient With Primary Pulmonary Meningioma With Associated Micro-Solid Nodules

**DOI:** 10.7759/cureus.15700

**Published:** 2021-06-16

**Authors:** Ritu Chakrabarti, Damanjit Ghuman

**Affiliations:** 1 Internal Medicine, Jersey City Medical Center, Jersey City, USA; 2 Hematology and Medical Oncology, Jersey City Medical Center, Jersey City, USA

**Keywords:** o primary pulmonary meningioma, incidentaloma, lung cancer, micronodule, solitary pulmonary nodule

## Abstract

Primary pulmonary meningiomas (PPMs) are rare mesodermal tumors that arise in the lung and are most often incidentally identified as single pulmonary nodules. Most cases of PPM are benign, and surgical resection remains the primary curative treatment. We describe the case of a 65-year-old asymptomatic female who presented with an incidentally identified 2.5 x 1.7-cm lobulated, non-calcified mass in the right lower lobe of the lung, which was diagnosed as PPM that had low fluorodeoxyglucose (FDG)-avidity and associated sub-centimeter nodules present in the same lobe. The patient was closely monitored and the nodules showed essentially no interval enlargement over several months. Given the disparate locations and small sizes of the nodules, no surgical resection was planned. The patient remained clinically stable, and close medical monitoring was determined to be the best course of action. Our case highlights the viability of medical monitoring as an alternative to surgery in asymptomatic patients with benign PPMs that have associated micronodules.

## Introduction

Meningiomas are the most common central nervous system tumors; however, they may also arise in extracranial sites such as the head, skin, and peripheral nerves. In rare instances, primary pulmonary meningiomas (PPMs) have been identified as ectopic meningiomas arising as primary tumors in the lung [1-2]. So far, fewer than 60 cases of this condition have been described, and most of them have been incidentally identified on CT and further characterized by positron emission tomography (PET) scan and subsequent biopsy [3]. While the majority of PPM cases are benign, malignant cases have also been reported. Treatment is often curative with wedge resection or pneumonectomy [4], and benign PPMs have an excellent prognosis. However, only a few cases in the literature have discussed the diagnosis and treatment of benign PPMs with multiple associated nodules to date. In this case report, we discuss the level of complexity associated with the diagnosis and management of a benign case of PPM with faint fluorodeoxyglucose (FDG)-avidity and associated micro-solid nodules.

## Case presentation

**Figure 1:**
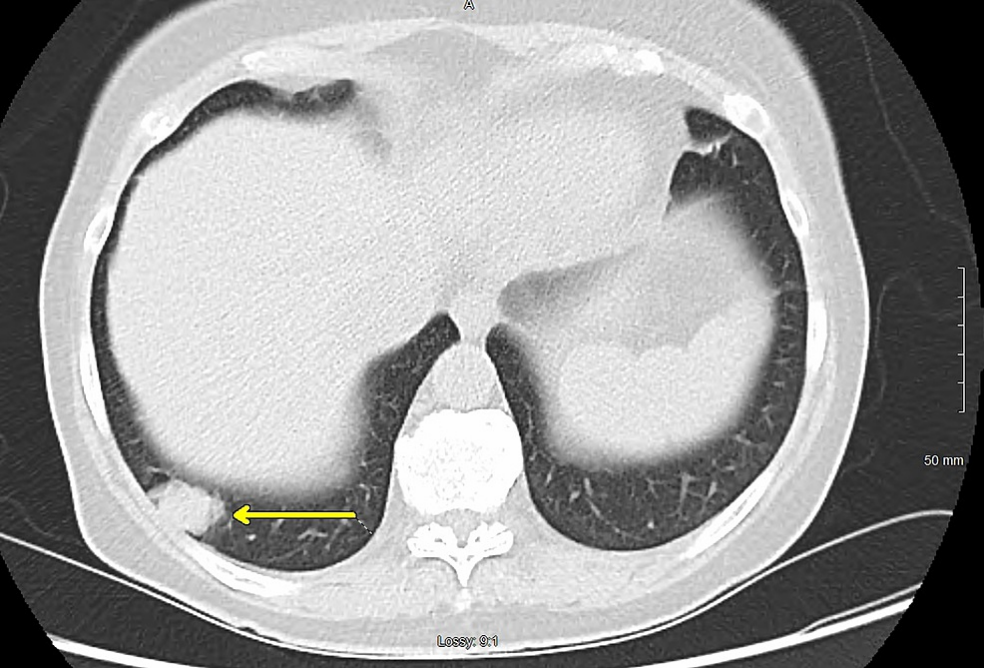
CT scan in the axial view demonstrating a non-calcified, lobulated mass (arrow) in the right costophrenic angle CT: computed tomography

**Figure 2:**
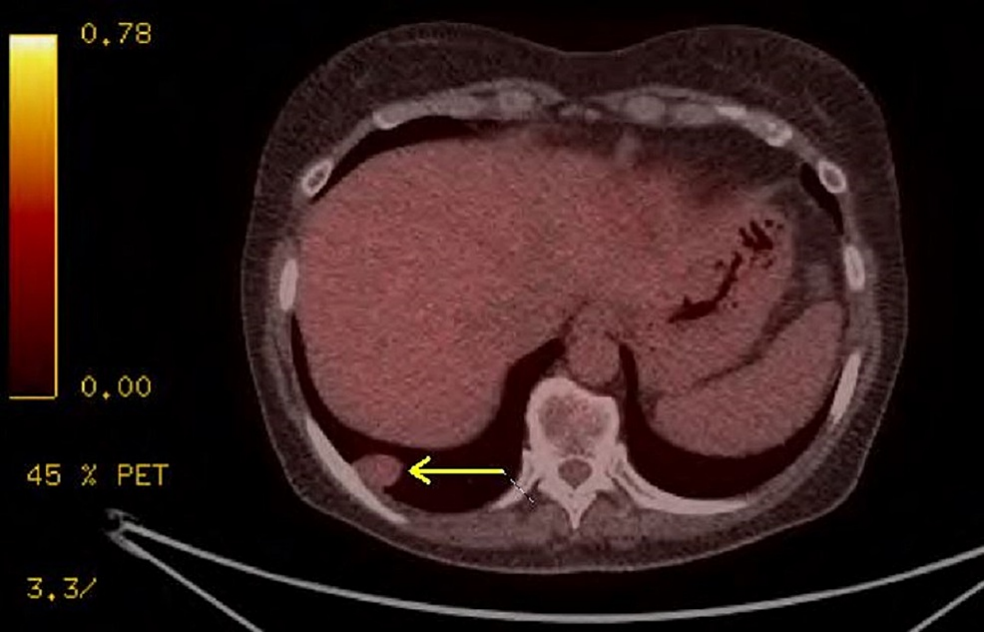
CT/PET of the chest in the axial view demonstrating a 2.5 x 1.7-cm lobulated non-calcified mass (arrow) in the right posterior sulcus with faint FDG-avidity of SUVmax of 2.127 CT: computed tomography; PET: positron emission tomography; FDG: fluorodeoxyglucose; SUVmax: maximum standardized uptake value

**Figure 3:**
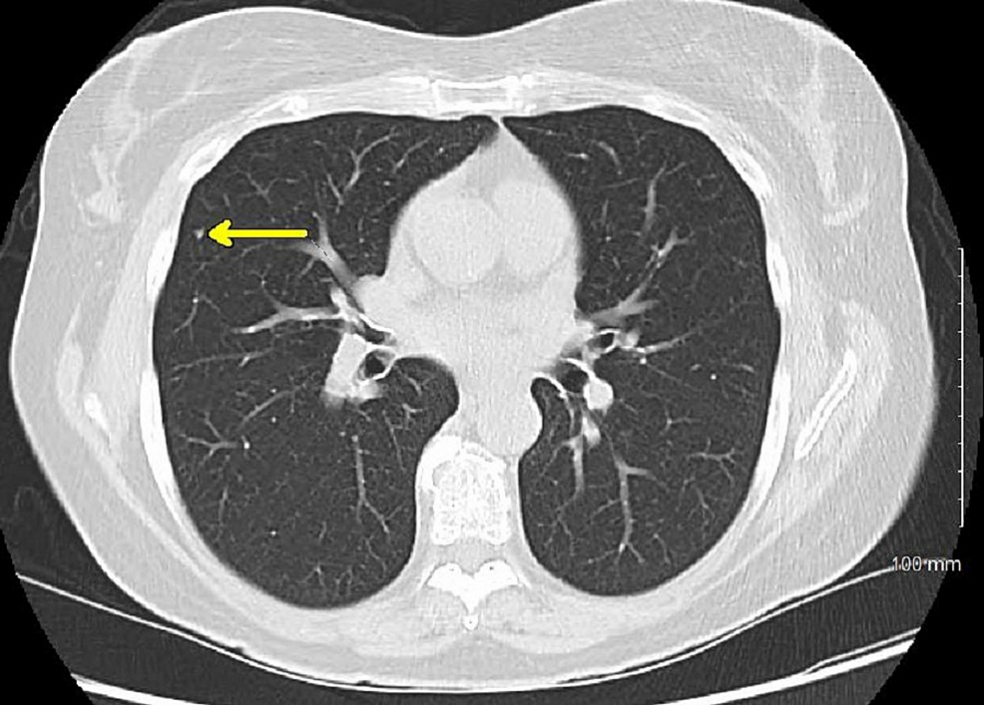
CT scan in the axial view demonstrating sub-centimeter nodule (arrow) in the right upper lobe CT: computed tomography

**Figure 4:**
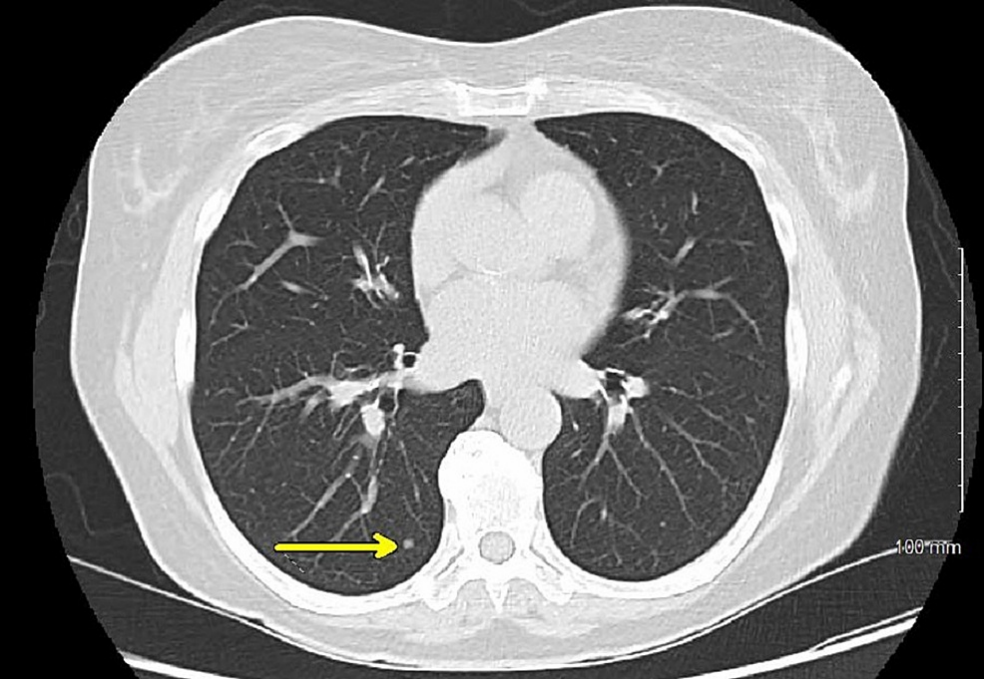
CT scan in the axial view demonstrating sub-centimeter nodule (arrow) in the right middle lobe CT: computed tomography

A 65-year-old female with no significant past medical history was concerned about her family history of cardiac disease and requested to undergo imaging for a coronary artery calcification (CAC) score. The patient had a five-pack-year smoking history and had quit 10 years prior; she was otherwise healthy without any symptomatic complaints. Non-contrast CT of the chest did not reveal identifiable cardiovascular disease but did reveal an incidental right lung nodule. Repeat CT scan and PET scan revealed a 2.5 x 1.7-cm lobulated non-calcified mass in the right posterior sulcus with faint FDG-avidity of maximum standardized uptake value (SUVmax) of 2.127 (Figures 1, 2), an 8.4-mm elliptical translucent nodular opacity, and additional nodules throughout the right lung that were smaller than 3 mm with insignificant FDG-avidity (Figures 3, 4).

**Figure 5:**
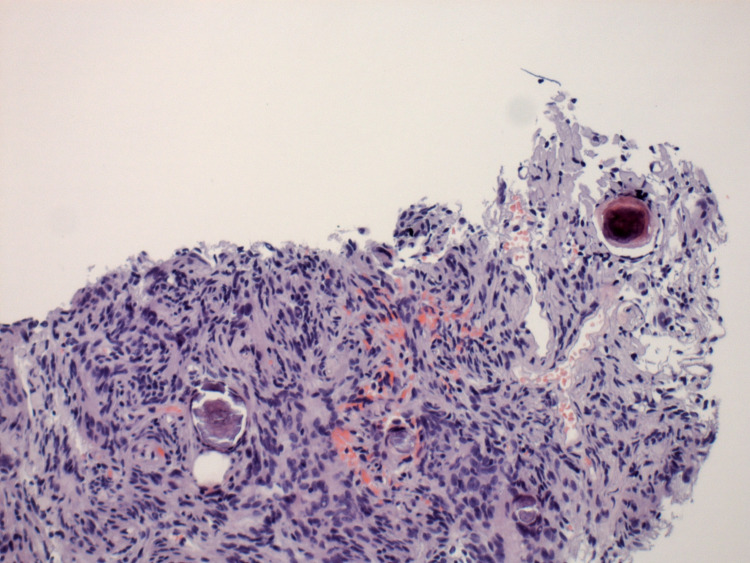
Biopsy of the lung mass demonstrating psammoma bodies amidst spindle cell lesions in whirling patterns (H&E 10x) H&E: hematoxylin and eosin

**Figure 6:**
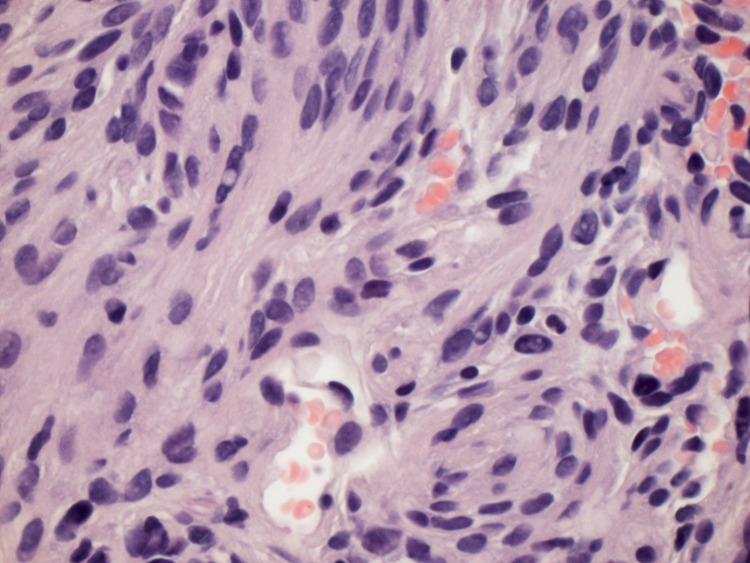
Biopsy of the lung mass demonstrating uniform spindle cells with a whirling pattern (H&E 40x). Cytological atypia is mild, mitosis is rare, and there is no necrosis H&E: hematoxylin and eosin

Lymph nodes did not demonstrate abnormal metabolism. CT-guided lung biopsy of the right lower lobe posterior mass was subsequently performed and revealed a grossly soft, tan specimen with spindle cell lesions, focal whirling pattern, and presence of psammoma bodies (Figure 5). Cytologically atypical cells were mild, mitosis was rare, with no necrosis (Figure 6). Immunohistochemistry showed focally positive results for CD-34, factor 13A, P63, D2-40, and epithelial membrane antigen (EMA), with Ki67 at 1%. The lesion was diagnosed as a benign pulmonary meningioma, and the patient underwent a repeat PET scan seven months later, which revealed largely unchanged findings with slight interval enlargement of a 4-mm non-calcified micronodule in the right lower lobe. The patient remained asymptomatic and was scheduled for a follow-up in six months.

## Discussion

Since the first case report published in 1983 by Kemnitz et al. [5], 53 known cases of PPM have been described in the literature, with 40 cases being confirmed by biopsy [3]. PPMs are most often incidental findings in patients between the ages of 40-60 years, with a slight predilection for females [3]. The pathogenesis of PPM remains unknown; current hypotheses include association and growth from meningothelial-like nodules [6] as well as multifunctional mesenchymal cells persistent in the lung [7]. Patients are typically asymptomatic even though some have respiratory symptoms including dyspnea, hemoptysis, or cough. Imaging on X-ray and CT scans typically reveals a round or oval solitary pulmonary nodule with smooth boundaries ranging between 0.6-6 cm in size; however, three malignant tumors ranging between 5-15 cm have been previously described in the literature [8-9].

Histopathologically, PPMs can be categorized as epithelial, fibrous, or, most commonly, transitional, and they are typically characterized by the presence of spindle cells in whirlpool arrangements and psammoma bodies. Cytology shows the expression of vimentin, EMA, and CD-34 positivity and S-100 and keratin negativity [10-11]. While a few malignant cases have been documented as relapsing despite receiving treatment [10], most nodules are benign, well-circumscribed, and solitary; consequently, surgical intervention through wedge resection or lobectomy are the main treatment modalities. Sub-centimeter micronodules have also been diagnosed as PPM [12]; however, they are surgically difficult to resect.

We described a rare case of an incidentaloma diagnosed as PPM with associated micronodules. Our patient had a 2.5-cm mass in the right lower lobe, showing relatively faint FDG-avidity with SUVmax of 2.127, which was a biopsy-proven PPM with additional smaller micronodules in the right upper and lower lobe with insignificant FDG-avidity. There is a high likelihood that these micronodules may also represent PPMs as there was a slight interval enlargement of a 4-mm nodule; furthermore, pulmonary tumors have been documented to have low FDG-avidity and still have a 24% chance of malignancy [13]. Although surgical resection is the mainstay of treatment in diagnosed PPMs, the location and size of the smaller nodules in our patient made them inaccessible for biopsy or resection. Additionally, the patient remained asymptomatic and the biopsy-proven 2.5-cm mass was unchanged on follow-up seven months later. Consequently, we determined that a conservative approach of close medical monitoring would be the best option for our patient.

## Conclusions

PPMs are relatively rare causes of incidental solitary pulmonary nodules, and are typically well-circumscribed soft tumors with histopathology displaying spindle cells in whirlpool arrangements and psammoma bodies with vimentin, EMA, and CD-34 positivity. We discussed a case of a patient who presented with these typical features of PPM along with associated sub-centimeter micronodules, all of which showed relatively low or faint FDG-avidity; to date, only a few case reports have described PPM with micronodules. Given the location and distance between the primary tumor and smaller nodules, an extensive discussion regarding the management of the nodules was undertaken, which led to the decision to opt for continued medical monitoring over surgical resection, which is the current standard of treatment for most benign PPMs. We highlight the importance of including PPM in the differential diagnoses of solitary pulmonary nodules and micro-solid nodules, along with a consideration for conservative management in asymptomatic patients with benign tumors.
